# Complexity, connectivity, and duplicability as barriers to lateral gene transfer

**DOI:** 10.1186/gb-2007-8-8-r156

**Published:** 2007-08-02

**Authors:** Alon Wellner, Mor N Lurie, Uri Gophna

**Affiliations:** 1 Department of Molecular Microbiology and Biotechnology, George S Wise Faculty of Life Sciences, Tel Aviv University, Tel Aviv, Israel, 69978

## Abstract

**Background:**

Lateral gene transfer is a major force in microbial evolution and a great source of genetic innovation in prokaryotes. Protein complexity has been claimed to be a barrier for gene transfer, due to either the inability of a new gene's encoded protein to become a subunit of an existing complex (lack of positive selection), or from a harmful effect exerted by the newcomer on native protein assemblages (negative selection).

**Results:**

We tested these scenarios using data from the model prokaryote *Escherichia coli*. Surprisingly, the data did not support an inverse link between membership in protein complexes and gene transfer. As the complexity hypothesis, in its strictest sense, seemed valid only to essential complexes, we broadened its scope to include connectivity in general. Transferred genes are found to be less involved in protein-protein interactions, outside stable complexes, and this is especially true for genes recently transferred to the *E. coli *genome. Thus, subsequent to transfer, new genes probably integrate slowly into existing protein-interaction networks. We show that a low duplicability of a gene is linked to a lower chance of being horizontally transferred. Notably, many essential genes in *E. coli *are conserved as singletons across multiple related genomes, have high connectivity and a highly vertical phylogenetic signal.

**Conclusion:**

High complexity and connectivity generally do not impede gene transfer. However, essential genes that exhibit low duplicability and high connectivity do exhibit mostly vertical descent.

## Abstract 2

Laterally transferred genes are shown to be less involved in protein-protein interactions, and essential genes that exhibit low duplicability and high connectivity do exhibit mostly vertical descent.

## Background

**Figure 1:**
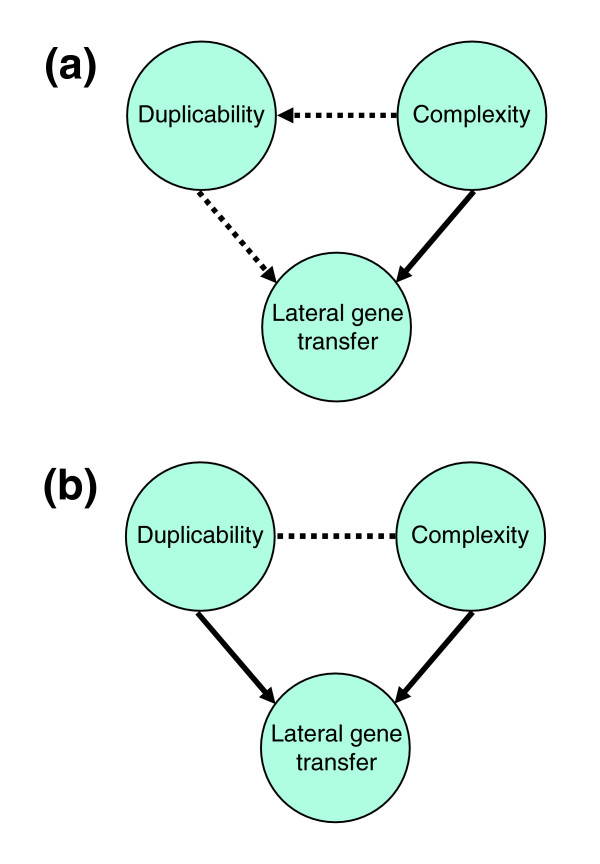
Alternative scenarios for the effect of complexity and duplicability on lateral gene transfer. **(a) **Complexity could be operating directly (solid line) on LGT or indirectly (dotted line) through its effect on duplicability; **(b) **Complexity and duplicability are correlated (dotted line), and each is effecting LGT independently.

Lateral gene transfer (LGT) is a major force in microbial evolution, driving bacterial genetic innovation and speciation [1,2]. The common intuitive notion of a lateral transfer event is an acquisition of a locus or allele with a new and potentially useful function. Indeed, it has been claimed that laterally acquired genes may only be fixed in a population if they are under strong positive selection [3]. The scarcity of transfer of genes involved in informational processes ('informational genes') such as transcription and translation was, therefore, attributed to lack of positive selection due to the inability of newly acquired proteins to interact with their pre-existing native counterparts [4]. According to this concept, designated 'the complexity hypothesis' [4], the chances of a gene to be beneficial to a new host are greatly influenced by the number of its interactions with its new neighbors - implying a direct link between complexity and LGT (Figure 1a, solid arrow).

An alternative explanation for the paucity of transfer of informational genes may be negative (purifying) selection operating against gene acquisition. For many major cellular functions, in particular essential ones, there is likely to be a homologous ancestral gene already present when a foreign ortholog is acquired. In these cases, the laterally acquired gene will have to coexist alongside its native homolog before orthologous replacement (also called xenologous gene displacement) can occur. If the foreign gene is expressed, the chances that this coexistence will be deleterious to the host is especially high when the gene's encoded protein is involved in protein-protein interactions, and even more so when it is a subunit of a protein complex. This is because the increase in concentration of one component (that is, the effect of gene dosage resulting from a single gene duplication) can either inhibit complex assembly, or form undesirable toxic interactions, as described in 'the balance hypothesis' [5]. The authors showed that genes encoding protein complex subunits have lower duplicability, that is, are less likely to have multiple paralogs in a genome. We propose that a horizontally acquired homolog can exert an even more profound negative influence, compared with a duplication product, even at low expression levels. This is because it could be similar enough to interact with a native protein and yet sufficiently different so that an aberrant interaction is formed, destabilizing a native complex in what is often called a 'dominant negative' effect. This situation will result in an indirect effect of complexity on LGT mediated through gene duplicability (Figure 1a, dotted arrows).

Here we perform several analyses aimed at testing the compatibility of the complexity hypothesis and the balance hypothesis with existing protein interaction data, using *Escherichia coli *as a model for prokaryotic evolution.

## Results and discussion

### Protein complexity and duplicability in *E. coli*

Previous work in the yeast *Saccharomyces cerevisiae *demonstrated that protein complexity (number of subunits in a protein) and gene duplicability are inversely correlated, which was attributed to the effects of gene dosage [5]. Accordingly, members of large gene families (having at least three paralogs) were shown to be significantly under-represented in yeast protein complexes when compared to singletons [5]. We tested whether a similar trend exists in the model prokaryote *E. coli*, which is somewhat less complex than yeast on the organismal level. All available subunit data for *E. coli *were retrieved from SwissProt [6], and family membership for each gene was obtained from the EMU server (see Materials and methods). The comparison showed that, in *E. coli*, gene families with three or more paralogs are in fact under-represented in monomers (20.44%) and not, as expected, in hetero-oligomeric protein complexes, where their fraction (29.98%) is very similar to that of the whole genome (31.24%). Furthermore, the data indicated that unlike yeast [7], a group composed of characterized monomers and homo-oligomers had a similar fraction of singletons (Q) to that of the whole *E. coli *proteome, and monomers of *E. coli *even have a higher Q value than the genomic fraction (Table 1). Notably, whereas in *Saccharomyces *the Q value for hetero-oligomeric proteins of medium size and above (having at least three subunits of more than one type of polypeptide) was very high, the corresponding Q value in *E. coli *was not higher than that observed for monomers in the latter organism. Thus, current *E. coli *protein data do not support a link between gene duplicability and membership in protein complexes.

**Table 1:** Fraction of singletons (Q) for various subsets of protein-encoding *E. coli *genes

Source	Protein subset	Number of proteins	Number of singletons	Q
SwissProt	Monomers	137	87	0.6350
	Homo-oligomers + monomers	596	333	0.5587
	Hetero-oligomers	407	228	0.5602
	Mid-to-large complexes (homotrimer and above)	334	197	0.5898
	1 external interaction	641	342	0.5335
	>1 external interaction	125	75	0.5000
Arifuzzaman *et al*. 2006 [9]	1 external interaction	371	193	0.5202
	>1 external interaction	1,916	1,049	0.5475
Ragan 2001 [11]	LGT due to atypical nucleotide composition	568	339	0.5968
Beiko *et al*. 2005 [13]	LGT by Bayesian phylogenetic analysis	987	607	0.6150
PEC database	Essential genes	233	173	0.7425
	*E. coli *average	4,308	2,382	0.5529

The lack of correlation between protein complexity and duplicability in *E. coli *does not support the balance hypothesis for this organism, which may hint at different selective forces on complexity in prokaryotic genomes to those operating in eukaryotes. This finding is in agreement with recent findings by Ochman and colleagues [8], demonstrating differences in protein interaction network evolution between eukaryotes and prokaryotes.

Protein-protein interactions outside multimeric proteins also contribute greatly to the complexity of an organism. However, unlike subunits in a protein complex, external interactions usually do not require precise stoichiometry and may, consequently, be less sensitive to dosage effects. Therefore, one would expect these interactions not to be linked to duplicability. Indeed, there was no significant difference in Q for proteins having a single interaction versus those with multiple interactions in *E. coli *(*p *= 0.21). Recently, a high-throughput analysis of protein-protein interactions in *E. coli *has been carried out using a His-tagged clone library pull-down [9] that is more sensitive than the TAP- or SPA-tagged bait protein approach used previously [10] and thus provides interaction data for most *E. coli *proteins. High-throughput methods cannot distinguish between interactions of subunits within a stable protein complex and those that are external to the complex. Therefore, the data include the cumulative contribution of both types of interaction to complexity. This category of combined interactions will henceforth be referred to as connectivity. In agreement with the SwissProt data, the Q value of proteins with more than one interaction in the pull-down study (Table 1) was not significantly higher than that of proteins with a single interaction (*p *= 0.71). Nevertheless, an analysis of the number of interacting partners of these proteins showed that, in *E. coli*, singletons have significantly more partners (a higher connectivity) than do proteins with paralogs (4.474 versus 4.095, *p *= 0.01), so some correlation between connectivity and duplicability does exist.

### Characterized protein complexes are not resistant to transfer

Correlating complexity or duplicability with LGT on a genomic scale is complicated by the fact that different LGT detection methods often identify different subsets of genes [11,12]. Therefore, for our analysis we relied on two different datasets: genes identified as being acquired by LGT by Bayesian phylogenetic analysis [12,13] and genes identified as transferred due to atypical nucleotide composition [11,14]. This last group of genes is thought to represent relatively recent transfer events [11,12]. In order to address general 'transferability' of genes in microbial evolution, rather than just specific origin of genes in *E. coli*, we used an established global estimator, the 'phylogenetically discordant sequence' (PDS) metric. This parameter measures the extent to which a protein's phylogenetic signal matches most other proteins' phylogenetic signals in a genome by examining its similarity to its reciprocal best matches in other genomes [15,16]. Values range from 0 to 1, where a totally concordant sequence has a score of 1, and a highly discordant protein has a score of 0. It is important to note that a gene that is vertically derived in *E. coli *but has been involved in many LGT events in other taxa could have a low PDS score, due to its irregular pattern, which is appropriate for global assessment of a gene's propensity for LGT. Also, transfers within closely related organisms will generally affect PDS to a lesser extent than transfers between remote taxa [15].

Having a foreign variant of a protein could have a destabilizing effect on a complex, even a homo-oligomeric one, resulting in a dominant negative phenotype. If selection against LGT is mediated by such a dominant negative mechanism, one would expect to observe a difference between the LGT propensity of monomers, which should be free of such selection, and oligomeric complexes, such as homodimers or homotetramers. When comparing *E. coli *proteins (Table 2) with SwissProt subunit data (see Materials and methods) we observed no significant difference in PDS between monomers and other homo-oligomers (*p *= 0.056), indicating no support for such a role in bacterial protein evolution. When a protein complex involves more than one type of polypeptide, the chance for a negative influence of a foreign homolog may increase due to the effect of gene dosage - the requirement of a precise molar ratio between subunits in order to guarantee a functional complex [5]. However, comparing PDS values between hetero-oligomeric complexes and the group containing monomers/homo-oligomers showed only an insignificant increase in the average PDS value (*p *= 0.16). It appears, therefore, that protein complexes are seldom a major barrier to transfer, unless essential complexes are involved (see below). A possible explanation for this observation is that genes encoding complex components tend to be located adjacent to each other in prokaryotes. Thus, a lateral transfer event can, in principle, transfer the whole complex as a unit [17], be it on a plasmid or genomic island. A good example of an extremely large and complex structure that has been frequently transferred is the virulence-associated bacterial type III secretion systems [18]. Similarly, a global survey of LGT across microbial genomes has shown microbial surface structures such as pili, which are often multimeric, to be frequent products of transfer [19]. Pili-encoding genes are nearly always found in operons, so the combination of a function that improves fitness in a niche and location appears to be more potent than the negative effects of complexity, if any, in determining transferability.

**Table 2:** Mean phylogenetic discordant sequence score for different subsets of *E. coli *proteins

Group	Number of proteins	Mean PDS (SEM)	Pair-wise significance of comparison (*p*)
Monomers	137	0.807 (0.028)	0.056
Homo-oligomers	459	0.758 (0.017)	
Monomers + homo-oligomers	596	0.769 (0.015)	0.164
Hetero-oligomers	407	0.794 (0.018)	
Singletons	2,382	0.739 (0.007)	<0.00001
Non-singletons	1,920	0.678 (0.009)	
1 interaction (SP*)	150	0.766 (0.029)	0.00005
>1 interaction (SP)	641	0.845 (0.013)	
1 interaction (PD^†^)	368	0.732 (0.02)	0.086
>1 interaction (PD)	1,897	0.711 (0.009)	0.086

* Interaction subsets based on SwissProt data. ^†^Interaction subsets based on pull-down data. SEM, standard error of the mean.

### Frequently transferred genes have fewer external interaction partners

Although the original complexity hypothesis was mostly focused on protein complexes, the authors nevertheless left room for other interactions. Indeed, Jain and colleagues [4] stated that "... the probability of a successful horizontal transfer will be strongly affected by the number of interactions that a protein must make with its neighbors." Thus, we suggest that the scope of the complexity hypothesis should be expanded to include all connectivity, rather than complexity. Characterized *E. coli *proteins that were found to be involved in a single interaction were, therefore, compared to proteins with multiple interactions (Table 2). Notably, proteins with multiple interactions have a higher average PDS value (0.845) than proteins with a single interaction (0.766), and the difference is significant (*p *= 0.00005). A similar trend was observed for the pull-down data, but was not significant (PDS scores of 0.732 and 0.711, respectively, *p *= 0.086). Thus, genes that are more frequently transferred in evolution tend to have lower connectivity, in agreement with our broader definition of the complexity hypothesis. The fractions of laterally transferred interacting genes (one interaction or more) in *E. coli*, identified by either composition or Bayesian phylogeny, were not significantly different from the average for *E. coli*. However, high-throughput interaction data (that includes interactions within a protein complex, see above) indicate that although transferred genes identified by Bayesian phylogeny have a higher connectivity average that is not significant (4.495 versus the *E. coli *average of 4.305, *p *= 0.311), the acquired genes with atypical composition, assumed to be more recent arrivals in the genome, have a significantly lower number of interactions (3.9922, *p *= 0.049). It therefore appears that genes that are more recent arrivals in a genome have lower connectivity than the rest of the genes and probably have not integrated fully into the genome's interaction network. Thus, the broader sense of the complexity hypothesis is again in agreement with the data. The evolutionary mechanism behind our observation remains unclear - is it that genes that have to interact with multiple partners are seldom retained or do transferred genes just gradually adapt to the new network? We feel our findings regarding recently transferred genes support the latter explanation, but the former alternative cannot altogether be rejected.

### Duplication resistant genes are also resistant to LGT

We compared the PDS values in the *E. coli *genome of single copy genes (singletons) to those of genes that are part of gene families or have at least one paralog (Table 2). Singletons have a significantly higher average PDS value than genes with paralogs (*p *< 0.00001), indicating that genes that are seldom duplicated are also rarely transferred, correlating duplicability with transferability. Conversely, putative transferred genes of atypical composition in *E. coli *have a high singleton fraction, exceeding the average singleton content of *E. coli*, though this was not statistically significant (0.597 versus 0.553, *p *= 0.053). While this finding may seem contradictory, it is not surprising, given that many genes with atypical composition are either ORFans [20] or very poorly conserved singletons. The other dataset of putative transfers could not be used for comparison since it is methodically enriched for singletons [13]. A possible explanation is that xenologous gene displacement is more likely to occur when the replaced allele already has paralogs in the genome. Indeed, genes that have three paralogs or more in *E. coli *had significantly lower PDS values than those where only a pair of paralogs exists (0.66 versus 0.722, *p *= 0.00004).

Surprisingly, low duplicability by itself (that is, not reflecting the effect of complexity; Figure 1) seems to operate as a barrier to transfer, even though complexity does not (see above). This finding indicates that other forces may be at play, such as the ability of the cell to maintain optimal metabolic fitness in the presence of an extra homolog that may disrupt its regulatory homeostasis. Paradoxically, a gene acquired from a genetically remote organism may be less harmful to the organism, since it is less likely to be expressed at a significant level, owing to poor promoter recognition by the resident RNA polymerase sigma factors and alien codon usage. Indeed, artificially implanting the genome of *Synechocystis PCC6803 *into the genetically distant bacterium *Bacillus subtilis *was successful once the former's rRNA operons were removed, thus probably reducing the expression levels of alien proteins [21]. Furthermore, a closely related allele may be homogenized by gene conversion through homologous recombination, while a distant one cannot undergo this process [22]. Thus, duplicability barriers can on the one hand prevent LGT and on the other promote fixation of genes from remote sources.

### Essential genes and conserved singletons

To further establish a link between duplicability and LGT, we examined a collection of genes that were 'conserved singletons', meaning they were both ubiquitous and had no paralogs, in several gamma-proteobacteria [23], and observed that 200 out of 205 genes had PDS values greater than 0.99, reflecting probable vertical descent in most known genomes. This is in agreement with Lerat and colleagues' [23] finding, based on phylogenetic inference, establishing that 203 out of 205 singleton genes had compatible phylogenies within the gamma-proteobacteria. Thus, it is reasonable to conclude that genes that are conserved singletons will tend to be rarely transferred.

An unusually large fraction (74.25%) of essential protein-coding genes in *E. coli *are singletons. The trivial explanation is that for these genes no paralog can compensate for a mutation, making them indispensable. However, one can also argue that the importance of these genes and precise regulation of their expression hinders their duplication. Indeed, of the 233 essential protein coding genes in *E. coli*, nearly half (115) are found in Lerat and colleagues' conserved singleton dataset, supporting the possibility of duplication resistance [23]. The average PDS value for those essential genes was high (0.97) as was their connectivity (5.765 interactions in the pull-down study). Unlike the majority of *E. coli *genes, both the complexity hypothesis and the balance hypothesis are supported for this unique assemblage. Relative resistance to transfer can be expected for those genes that are both highly ubiquitous and duplication-resistant because the transition state in which both foreign and native alleles coexist could be deleterious, if not fatal. We also hypothesize that genes that are conserved singletons in the vast majority of available prokaryotic genomes, in which they occur, will have a high impact on the fitness of these organisms.

## Conclusion

Our findings shed new light on current paradigms on transferability and duplicability of genes in prokaryotes. We propose that duplicability and, to a lesser degree, connectivity, can directly affect the fixation of laterally transferred genes in prokaryotic genomes (Figure 1b). We expand the complexity hypothesis to include general connectivity and show that, in its strictest sense, it applies mostly to essential complexes.

Many phylogenetic studies base their analysis on single copy genes to avoid problems in discerning orthology. Based on the findings presented here it is likely that this practice in fact filters out many laterally transferred genes. While this may be desirable when reconstructing organismal phylogenies ('trees of life') based on a non-transferred core [23], it is highly inappropriate when assessing the impact of LGT on different genomes.

## Materials and methods

### Identification of singletons and gene families

A dataset of all 4,302 *E. coli *K12 MG 1655 proteins was retrieved from the EMU web service [24]. We identified 480 gene families using the 'Genome query for gene families' query with a BLAST threshold of e^-10^; 1,920 genes were obtained that belonged to gene families. Subtraction of these genes from the dataset of all protein-coding genes resulted in 2,382 singletons.

### Identification of subunits of protein complexes and external interactions

Protein complex and protein interaction data were automatically retrieved from SwissProt/TrEMBL and manually sorted. Complex subunit information (SwissProt field 'Subunit') was obtained for 1,003 genes, out of which 137 genes could unequivocally be classified as monomers, 459 as homo-oligomers and 407 as hetero-oligomers. In addition, all homo-oligomers that formed a homotrimer or more complex structure were grouped as mid-to-large complexes. External protein interaction data (SwissProt field 'Interaction') were similarly retrieved from SwissProt. High-throughput interaction data were retrieved from a recent *E. coli *pull-down study [9].

### Identification of essential genes

We obtained 232 protein-coding essential genes for *E. coli *K-12 from the PEC (profiling of the *E. coli *chromosome) website [25].

### Phylogenetically discordant sequences determination

The PDS metric for *E. coli *proteins [15] was determined using the 'Sorted lists of ORF characteristics' query of the EMU web service. PDS values are based on the data from 352 microbial genomes available in EMU in September 2006 (Additional data file 1).

### Statistical analysis

The SPSS statistics package version 12 (SPSS Inc., Chicago, IL, USA) was used. Significance scores for comparisons of fractions of singletons were determined using the chi-square test. Significance for comparisons of PDS values and interaction partner numbers were determined using the Mann-Whitney-Wilcoxon U test.

## Abbreviations

LGT = lateral gene transfer; PDS = phylogenetically discordant sequence.

## Authors' contributions

AW and UG designed the study, AW and MNL carried out analyses, and AW and UG wrote the manuscript.

## Additional data files

The following additional data are available with the online version of this paper. Additional data file 1 lists the genomes used for phylogenetic discordance analysis.

## Supplementary Material

Additional data file 1 Genomes used for phylogenetic discordance analysis. Click here for file
